# Targeting ApoE–KCC2 Signaling Rescues GABAergic Synaptic Dysfunction and Depression-like Behaviors in Mice

**DOI:** 10.34133/research.0746

**Published:** 2025-06-17

**Authors:** Chengyuan Xu, Jing Liu, Mengru Guo, Jia Wang, Xianbing Bai, Chenlei Zhang, Xinyue Luan, Huailong Pei, Huan Liu, Xinyou Lv, Xiangming Ye, Binliang Tang, Ming Chen

**Affiliations:** ^1^ Department of Pharmacology, School of Pharmaceutical Sciences, Anhui Medical University, Hefei 230032, Anhui, China.; ^2^ Center for Rehabilitation Medicine, Rehabilitation & Sports Medicine Research Institute of Zhejiang Province, Department of Rehabilitation Medicine, Zhejiang Provincial People’s Hospital, Affiliated People’s Hospital, Hangzhou Medical College, Hangzhou 310014, Zhejiang, China.; ^3^ Department of Psychology, School of Humanities and Social Sciences, University of Science and Technology of China, Hefei 230026, Anhui, China.

## Abstract

Apolipoprotein E (ApoE) has been implicated in neurodegenerative diseases; however, its function and underlying mechanisms in depression remain elusive. In this study, we employed chronic social defeat stress (CSDS) to establish a mouse model of depression and observed significantly reduced ApoE expression in the hippocampus. By leveraging ApoE knockout (*ApoE^−/−^*) and knockdown (ApoE-KD) mouse models, we demonstrated that ApoE deficiency induced depression-like behaviors, which were closely associated with impaired GABAergic synaptic transmission and down-regulation of ApoE receptors and K^+^–Cl^−^ cotransporter 2 (KCC2). In addition, we found an interaction between KCC2 and the ApoE receptor low-density lipoprotein receptor (LDLR) through coimmunoprecipitation analysis. Moreover, overexpression of ApoE or targeted activation of GABAergic neurons in the hippocampus significantly reversed depression-like behaviors in both CSDS-exposed and ApoE-KD mice. Lastly, treatment with KCC2 activators, CLP290 and CLP257, restored the expression levels of KCC2 and the GABA_A_R α1 subunit, significantly alleviating depression-like behaviors induced by CSDS or ApoE-KD. Together, our results elucidate the pivotal role of ApoE in the pathophysiology of depression and highlight the ApoE–KCC2 signaling pathway as a potential target for developing innovative antidepressant therapies.

## Introduction

Depression ranks among the most prevalent psychological disorders, particularly affecting young individuals with a strikingly high incidence rate [1]. The pathological mechanism of depression is highly complex, involving the interaction of multiple biological factors and environmental factors [2,3]. Clinically, it is characterized by impaired reward processing, memory decline, and cognitive impairment [4]. Despite extensive research, the pathological mechanisms of depression remain elusive, resulting in a dearth of effective preventive and therapeutic strategies [5]. Consequently, there is an urgent need to delve deeper into the causes of depression and identify novel therapeutic targets.

Apolipoprotein E (ApoE), a critical lipid transporter, is predominantly expressed in the liver and brain. In the central nervous system (CNS), ApoE interacts with adenosine triphosphate (ATP)-binding cassette transporter A1 proteins on neurons and glial cells to facilitate lipid transport, forming high-density lipoproteins that promote neuronal repair and remodeling; it plays a critical role in lipid metabolism and nervous system function [6]. By binding to neuronal low-density lipoprotein receptor (LDLR) and LDLR-related protein 1 (LRP1) [7], ApoE intricately regulates multiple key signaling pathways, including the extracellular regulated protein kinase, mitogen-activated protein kinase, and protein kinase B pathways, thereby modulating the metabolism of amyloid precursor protein and governing the transcription of synapse-associated genes [8,9]. Notably, aberrant ApoE expression has been implicated in the pathophysiology of neuronal dysfunction and dysregulation [10,11]. Recent studies have demonstrated that ApoE4 aggravates the depression-like behaviors of aging mice by down-regulating ATP levels. This reduction in ATP triggers the loss of GABAergic interneurons within the hippocampus, ultimately impairing γ-aminobutyric acid (GABA)-mediated inhibitory synaptic activity [12]. Although these studies underscore a strong association between ApoE and depression, the precise molecular mechanisms remain largely unknown.

Emerging evidence strongly suggests that GABA signaling within the brain plays a pivotal role in the pathophysiology of numerous mental disorders, including depression. At the core of depressive pathology lies the dysregulation of the glutamate–GABA homeostasis, typically manifested as elevated glutamate levels or insufficient GABA availability [13–15]. GABA, serving as the main inhibitory neurotransmitter within the brain, is responsible for upholding the excitation/inhibition (E/I) equilibrium in the CNS, and its dysfunction has been consistently linked to depression [16–20]. Clinical investigations have revealed a significant reduction in GABA levels in the cerebrospinal fluid and cerebral cortex of depressed patients [21], which coincides with impaired synaptic function [22]. Restoring neuronal GABA levels ameliorates affective and cognitive symptoms of depression [23]. Additionally, ApoE deficiency and the ApoE4 genetic variant impaired dendritic development in the mouse brain, resulting in reduced dendritic complexity and synaptic density [11,24,25]. However, the precise mechanisms of how ApoE modulates GABAergic synaptic transmission remain poorly understood.

In this study, we sought to elucidate the role of ApoE in the pathophysiology of depression. We found that susceptible (Sus) mice exposed to chronic social defeat stress (CSDS) exhibited depression-like behaviors, accompanied by a significant reduction in ApoE and K^+^–Cl^−^ cotransporter 2 (KCC2) proteins in the hippocampus. Notably, overexpression of ApoE in the hippocampus significantly ameliorated the depression-like behaviors in Sus mice. Conversely, hippocampus-specific knockdown of ApoE induced depression-like behaviors in mice, which were concurrently characterized by disrupted GABAergic synaptic transmission. Lastly, we employed chemogenetic and pharmacological approaches to investigate the underlying mechanisms by which ApoE regulates GABAergic transmission and depression-like behaviors. Overall, our findings provide new insights into the function of ApoE in the pathophysiology of depression and shed light on developing new interventions.

## Results

### Decreased GFAP and ApoE expression in the hippocampus of Sus group mice after CSDS exposure

**Fig. 1.**
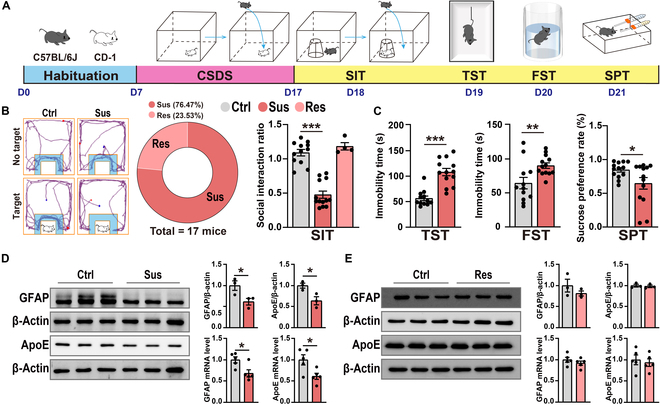
Decreased expression levels of glial fibrillary acidic protein (GFAP) and apolipoprotein E (ApoE) in the hippocampus of susceptible (Sus) group mice after chronic social defeat stress (CSDS) exposure. (A) Experiment flowchart and timeline for habituation, CSDS, and behavioral tests (social interaction test [SIT], tail suspension test [TST], forced swimming test [FST], and sucrose preference test [SPT]). (B) Representative tracks from the SIT (the blue area is marked as the social interaction [SI] zone) and a pie chart showing the proportion of Sus (social interaction ratio [SIR] < 1) and resilient (Res) (SIR > 1) mice (Sus, *n* = 13; Res, *n* = 4). Statistical results of SIR (control [Ctrl], *n* = 12; Sus, *n* = 13; Res, *n* = 4). (C) Statistical results of immobility time in the TST and FST (Ctrl, *n* = 12; Sus, *n* = 13). Statistical results of the SPT showing the sucrose preference rate (Ctrl, *n* = 13; Sus, *n* = 13). (D) Representative western blot bands for GFAP and ApoE in the hippocampus, with statistical results for GFAP and ApoE protein (Ctrl, *n* = 3; Sus, *n* = 3) and messenger RNA (mRNA) levels (Ctrl, *n* = 5; Sus, *n* = 5). (E) Representative western blot bands for GFAP and ApoE in the hippocampus, with statistical results for GFAP and ApoE protein (Ctrl, *n* = 3; Res, *n* = 3) and mRNA levels (Ctrl, *n* = 5; Res, *n* = 5). Values are presented as mean ± standard error of the mean (SEM). *P < 0.05; **P < 0.01; ***P < 0.001.

To investigate the role of ApoE in the pathophysiology of depression, we employed the CSDS model, a well-established paradigm for studying depression-like behaviors in rodents [26,27]. C57BL/6J mouse were exposed into the cage of a novel CD-1 mouse for 5 to 10 min of physical confrontation, followed by 24-h sensory contact through a perforated transparent acrylic divider. To prevent habituation, a different CD-1 mouse was introduced daily throughout the 10-d stress paradigm (Fig. 1A). We employed the social interaction test (SIT) to classify behavioral phenotypes in mice exposed to the CSDS model [28,29]. Following standardized screening procedures, compared with the control group (without exposure), stress-Sus mice exhibiting social avoidance behavior (social interaction ratio [SIR] < 1) were included in subsequent experiments. This phenotyping strategy ensured that subsequent depression-like behavioral tests were conducted on a well-defined population of mice displaying a clear depressive phenotype (Fig. 1B). Sus group mice exhibited a significant increase in immobility time during the forced swimming test (FST) and tail suspension test (TST) and a significant decrease in the sucrose preference rate during the sucrose preference test (SPT) compared with the control group (Fig. 1C). Additionally, we evaluated glial fibrillary acidic protein (GFAP, reactive astrocyte indicator) along with ApoE messenger RNA (mRNA) and protein levels in the hippocampus of both Sus and resilient (Res) group mice. It is noteworthy that astrocytes are the primary ApoE-producing cells in the CNS under physiological conditions [30]. The GFAP and ApoE mRNA and protein levels in the hippocampus of Sus group mice were significantly lower than those in the control group, whereas no such changes were observed in Res group mice (Fig. 1D and E). The number of GFAP+ cells was also significantly reduced in the hippocampus of Sus group mice compared with that in the control group (Fig. S1B). However, after CSDS exposure, there were no significant alterations in the expression levels of GFAP and ApoE in the medial prefrontal cortex (mPFC) and nucleus accumbens (NAc) of Sus group mice (Fig. S1C and D). Immunofluorescence labeling confirmed the co-occurrence of GFAP+ and ApoE+ cells (Fig. S2). Together, these findings suggest that the reduction in ApoE from hippocampal astrocytes may contribute to CSDS-induced depression-like behaviors.

### Overexpression of ApoE in the hippocampus alleviates depression-like behaviors induced by CSDS exposure

**Fig. 2.**
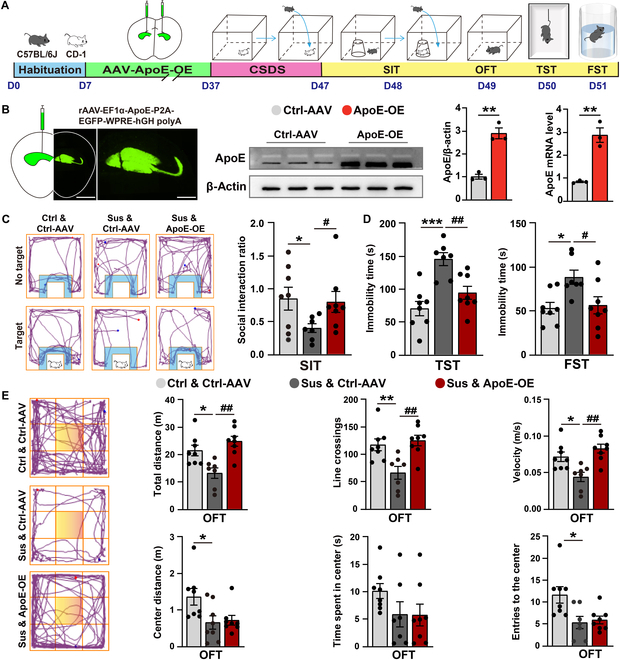
Overexpression of ApoE in the hippocampus alleviates depression-like behaviors induced by CSDS exposure. (A) Experiment flowchart and timeline for habituation, adeno-associated virus (AAV) injection, CSDS, and behavioral tests (SIT, open-field test [OFT], TST, and FST). (B) Expression of rAAV-EF1α-ApoE-P2A-EGFP-WPRE-hGH polyA in the hippocampus. Scale bar = 200 μm; enlarged image scale bar = 100 μm. Confirmation of ApoE protein and mRNA expression validates the overexpression efficacy of AAV-ApoE-OE (Ctrl-AAV, *n* = 3; ApoE-OE, *n* = 3). (C) Representative tracks from the SIT (the blue area is marked as the SI zone) and statistical results of the SIR (Ctrl & Ctrl-AAV, *n* = 8; Sus & Ctrl-AAV, *n* = 7; Sus & ApoE-OE, *n* = 8). (D) Statistical results of immobility time in the TST and FST (Ctrl & Ctrl-AAV, *n* = 8; Sus & Ctrl-AAV, *n* = 7; Sus & ApoE-OE, *n* = 8). (E) Representative tracks from the OFT (the yellow area is marked as the central zone) and statistical results of the distance moved, line crossings, speed, entries to the centers, and time/distance spent in the center during the OFT (Ctrl & Ctrl-AAV, *n* = 8; Sus & Ctrl-AAV, *n* = 7; Sus & ApoE-OE, *n* = 8). Values are presented as mean ± SEM. Ctrl & Ctrl-AAV vs. Sus & Ctrl-AAV, *P < 0.05, **P < 0.01, ***P < 0.001; Sus & Ctrl-AAV vs. Sus & ApoE-OE, ^#^*P* < 0.05, ^##^*P* < 0.01.

To further explore the role of ApoE in depression, we engineered an adeno-associated virus (AAV) vector designed to overexpress ApoE (ApoE-OE). Using stereotaxic surgery, we bilaterally delivered this ApoE-OE AAV into the hippocampus of C57BL/6J mice (Fig. 2A). Immunofluorescence analysis provided conclusive evidence of successful transfection, as vivid green fluorescence was observed within the hippocampal region (Fig. 2B). Quantitative analysis revealed that both the ApoE mRNA and protein levels in the ApoE-OE group increased by approximately 3-fold compared to those in the control vector (Ctrl-AAV)-expressed group (Fig. 2B). We pretreated mice with either Ctrl-AAV or ApoE-OE AAV, followed by exposure to CSDS. Subsequently, behavioral phenotyping was performed using the SIT to assess CSDS model mice (Fig. 2A). Behavioral assays demonstrated that ApoE overexpression had a profound impact on the depression-like behaviors of Sus mice. Specifically, compared to Sus mice treated with Ctrl-AAV, those with hippocampal ApoE overexpression exhibited a significantly increase in SIT and a significant reduction in immobility time in the TST and FST (Fig. 2C and D). Additionally, the open-field test (OFT) revealed a marked improvement in locomotor activity among Sus & ApoE-OE group mice compared with Sus & Ctrl-AAV group mice (Fig. 2E). Collectively, these results strongly indicate that hippocampal overexpression of ApoE exerts potent antidepressant effects.

### Hippocampal ApoE knockdown leads to GABAergic synaptic dysfunction and induces depression-like behaviors in mice

**Fig. 3.**
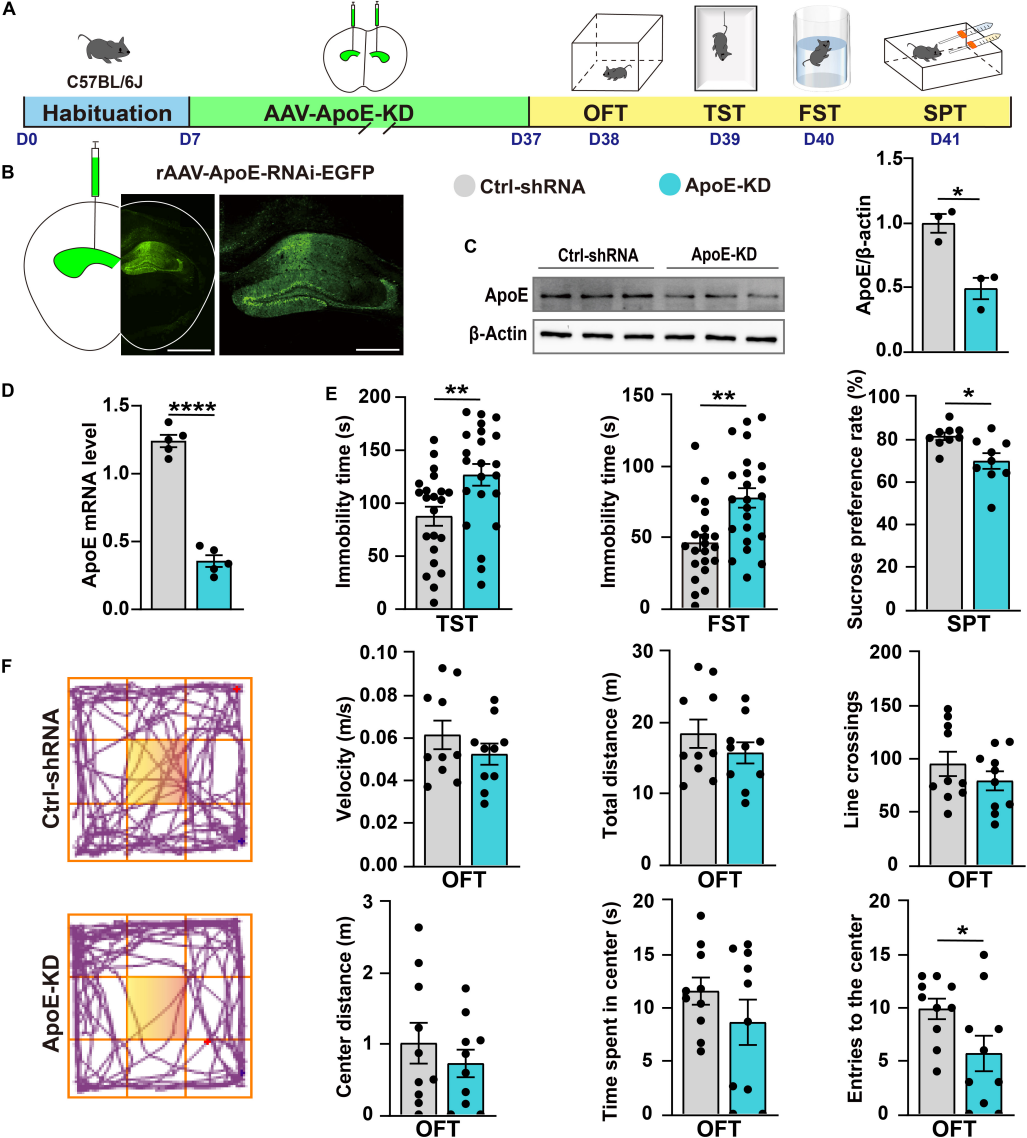
Hippocampal ApoE knockdown induces depression-like behaviors in mice. (A) Experiment flowchart and timeline for habituation, AAV injection, and behavioral tests (OFT, TST, FST, and SPT). (B) Expression of AAV-ApoE-KD in the hippocampus. Scale bar = 200 μm; enlarged image scale bar = 100 μm. (C) Confirmation of ApoE protein expression validates the knockdown efficacy of AAV-ApoE-KD (Ctrl-shRNA, *n* = 3; ApoE-KD, *n* = 3). (D) Confirmation of ApoE mRNA expression validates the knockdown efficacy of AAV-ApoE-KD (Ctrl-shRNA, *n* = 5; ApoE-KD, *n* = 5). (E) Statistical results of immobility time in the TST (Ctrl-shRNA, *n* = 22; ApoE-KD, *n* = 23) and FST (Ctrl-shRNA, *n* = 29; ApoE-KD, *n* = 30). Statistical results of the SPT showing sucrose preference rate (Ctrl-shRNA, *n* = 9; ApoE-KD, *n* = 9). (F) Representative tracks from the OFT (the yellow area is marked as the central zone). Statistical results of the distance moved, line crossings, speed, entries to the centers, and time/distance spent in the center during the OFT (Ctrl-shRNA, *n* = 10; ApoE-KD, *n* = 10). Values are presented as mean ± SEM. *P < 0.05; **P < 0.01; ***P < 0.001; ****P < 0.0001. ApoE-KD, ApoE knockdown; shRNA, short hairpin RNA.

**Fig. 4.**
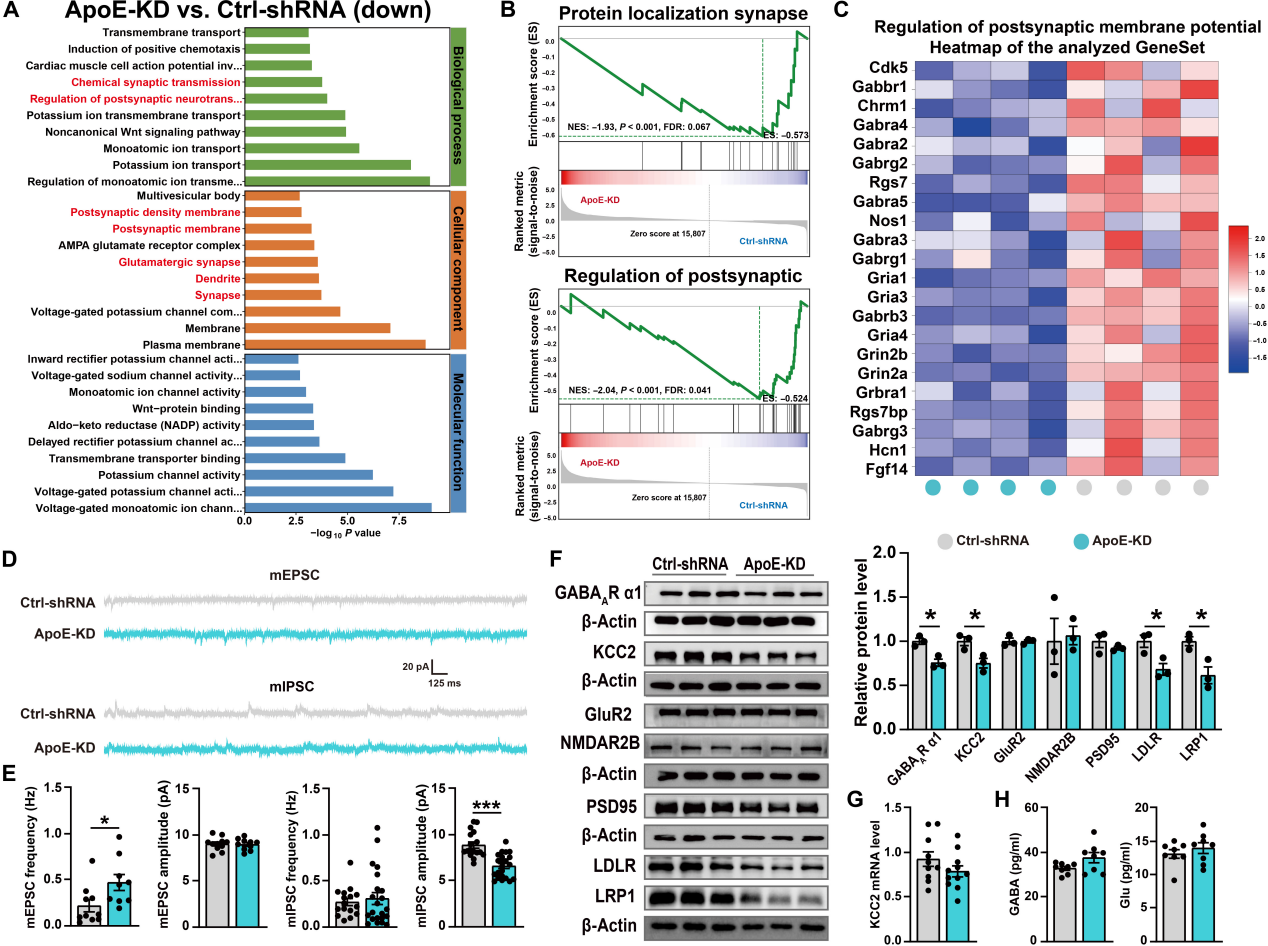
Hippocampal ApoE knockdown leads to synaptic dysfunction in mice. (A) Down-regulated genes after mouse hippocampal ApoE knockdown (Gene Ontology terms). (B) Results of gene set enrichment analysis (GSEA) for the transcripts in the trend analysis. (C) Regulation of the postsynaptic membrane potential heatmap of the analyzed GeneSet. (D) Representative traces of miniature excitatory postsynaptic current (mEPSC) and miniature inhibitory postsynaptic current (mIPSC) from the hippocampus. (E) Statistical results of mEPSC frequency (Ctrl-shRNA, *n* = 11 neurons from 3 mice; ApoE-KD, *n* = 10 neurons from 3 mice), mEPSC amplitude (Ctrl-shRNA, *n* = 10 neurons from 3 mice; ApoE-KD, *n* = 9 neurons from 3 mice), mIPSC frequency (Ctrl-shRNA, *n* = 17 neurons from 3 mice; ApoE-KD, *n* = 22 neurons from 3 mice), and mIPSC amplitude (Ctrl-shRNA, *n* = 16 neurons from 3 mice; ApoE-KD, *n* = 22 neurons from 3 mice). (F) Representative western blot bands for GABA_A_R α1, K^+^–Cl^−^ cotransporter 2 (KCC2), GluR2, NMDAR2B, PSD95, low-density lipoprotein receptor (LDLR), and LDLR-related protein 1 (LRP1) in the hippocampus, with statistical results for these proteins (Ctrl-shRNA, *n* = 3; ApoE-KD, *n* = 3). (G) Statistical results of KCC2 mRNA levels in the hippocampus (Ctrl-shRNA, *n* = 10; ApoE-KD, *n* = 10). (H) Enzyme-linked immunosorbent assay (ELISA) statistical results for γ-aminobutyric acid (GABA) and glutamate (Glu) levels in the hippocampus (Ctrl-shRNA, *n* = 8; ApoE-KD, *n* = 8). Values are presented as mean ± SEM. *P < 0.05; **P < 0.01; ***P < 0.001. NES, normalized enrichment score; FDR, false discovery rate.

To further validate the role of ApoE in depression-like behaviors, we employed an AAV-mediated short hairpin RNA (shRNA) strategy on C57BL/6J mice. We knocked down the ApoE expression in the bilateral hippocampus of 6-week-old mice (Fig. 3A). The precise localization of the AAV within the target brain region, along with the efficacy of ApoE gene silencing, was meticulously validated through immunofluorescence labeling, quantitative real-time polymerase chain reaction (qPCR) analysis, and western blot protein assays (Fig. 3B to D). Subsequent behavioral assessments revealed that ApoE-KD mice showed significantly increased immobility durations in the TST and FST. Additionally, these mice showed a significant decrease in the sucrose preference rate during the SPT (Fig. 3E). Notably, the running distance and time in the center of the OFT were not different from those of ApoE-KD mice (Fig. 3F), indicating that hippocampal ApoE knockdown selectively induced depression-like behaviors without affecting anxiety-like behaviors. To investigate the potential role of ApoE in depression, we performed RNA sequencing (RNA-seq) analysis on hippocampal tissues from both the Ctrl-shRNA and ApoE-KD groups. RNA-seq analysis revealed that hippocampal genes down-regulated following ApoE knockdown were significantly enriched in synaptic functions, particularly postsynaptic processes (Fig. 4A to C). These findings strongly suggest that hippocampus-specific ApoE knockdown results in synaptic dysfunction. Subsequently, we employed a slice patch clamp to record miniature excitatory postsynaptic current (mEPSC) and miniature inhibitory postsynaptic current (mIPSC) in CA1 pyramidal neurons. Notably, the amplitude of mIPSC was significantly decreased in the ApoE-KD group compared to that in the Ctrl-shRNA group (Fig. 4D and E). We further investigated the protein expression levels of excitatory (GluR2 and NMDAR2B) and inhibitory (GABA_A_R α1 subunit) postsynaptic receptors. In line with the mEPSC and mIPSC results, no significant changes were observed in the protein levels of excitatory receptors following hippocampal ApoE knockdown. In contrast, the protein level of GABA_A_R α1 subunit was significantly reduced (Fig. 4F). Additionally, enzyme-linked immunosorbent assay (ELISA) analysis revealed no significant differences in the levels of glutamate (Glu) and GABA in the hippocampus between the Ctrl-shRNA and ApoE-KD groups (Fig. 4H). Previous studies have indicated that the expression of GABA_A_R α1 is regulated by KCC2, a protein that governs the intracellular Cl^−^ gradient and facilitates GABAergic inhibition [31]. We quantified the mRNA and protein levels of KCC2 and observed a significant reduction in KCC2 protein levels after ApoE knockdown, while the mRNA levels remained unaltered (Fig. 4F and G). This result suggests that ApoE deficiency down-regulates KCC2 levels posttranscriptionally. ApoE has been shown to modulate downstream synaptic activity via ApoE-receptor-mediated pathways in neurons [8,9]. Given that both KCC2 and ApoE receptors are localized on the neuronal membrane surface, we investigated the expression levels of ApoE receptors. Western blot results showed a significant reduction in the levels of ApoE receptors, including LDLR and LRP1, following ApoE knockdown (Fig. 4F). Notably, reduced expression levels of KCC2, GABA_A_R α1, LDLR, and LRP1 were also detected in the hippocampus of Sus group mice (Fig. S1A). Collectively, these findings demonstrate that ApoE knockdown results in impaired GABAergic synaptic function and pronounced depression-like behaviors, which highlights the pivotal role of ApoE in regulating hippocampus-associated behaviors.

### Reduced LDLR, KCC2, and GABA_A_R α1 levels in *ApoE*^−/−^ mice

**Fig. 5.**
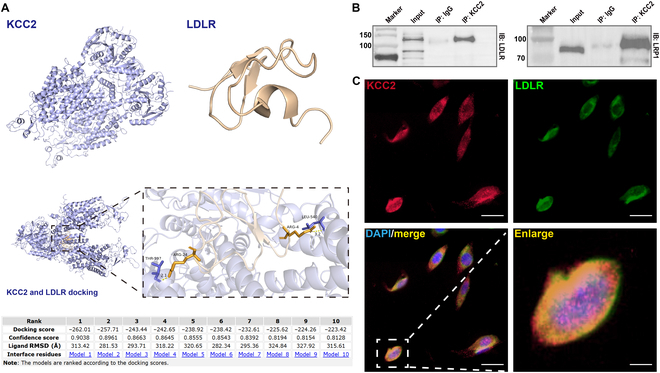
KCC2 interacts with ApoE receptors. (A) Three-dimensional structure of KCC2 and LDLR, 3-dimensional structure of KCC2 and LDLR docking, and molecular docking results of KCC2 and LDLR. (B) Coimmunoprecipitation (Co-IP) analysis showing the interaction between KCC2 and LDLR, as well as KCC2 and LRP1 in hippocampal lysates. (C) Representative fluorescence images of KCC2 and LDLR colocalization in HT22 cells. Scale bar = 20 μm; enlarged image scale bar = 5 μm. RMSD, root mean square deviation; DAPI, 4′,6-diamidino-2-phenylindole.

To elucidate the potential interactions between KCC2 and ApoE receptors, we employed molecular docking analysis. The results unveiled a specific hydrogen bond interaction between KCC2 and LDLR (Fig. 5A). Furthermore, coimmunoprecipitation experiments demonstrated protein–protein interactions between KCC2 with LDLR and LRP1 (Fig. 5B). Considering that KCC2 and LDLR are predominantly expressed on neuronal membranes, we conducted immunofluorescence staining in HT22 cells, which clearly demonstrated the colocalization of KCC2 with LDLR (Fig. 5C).

**Fig. 6.**
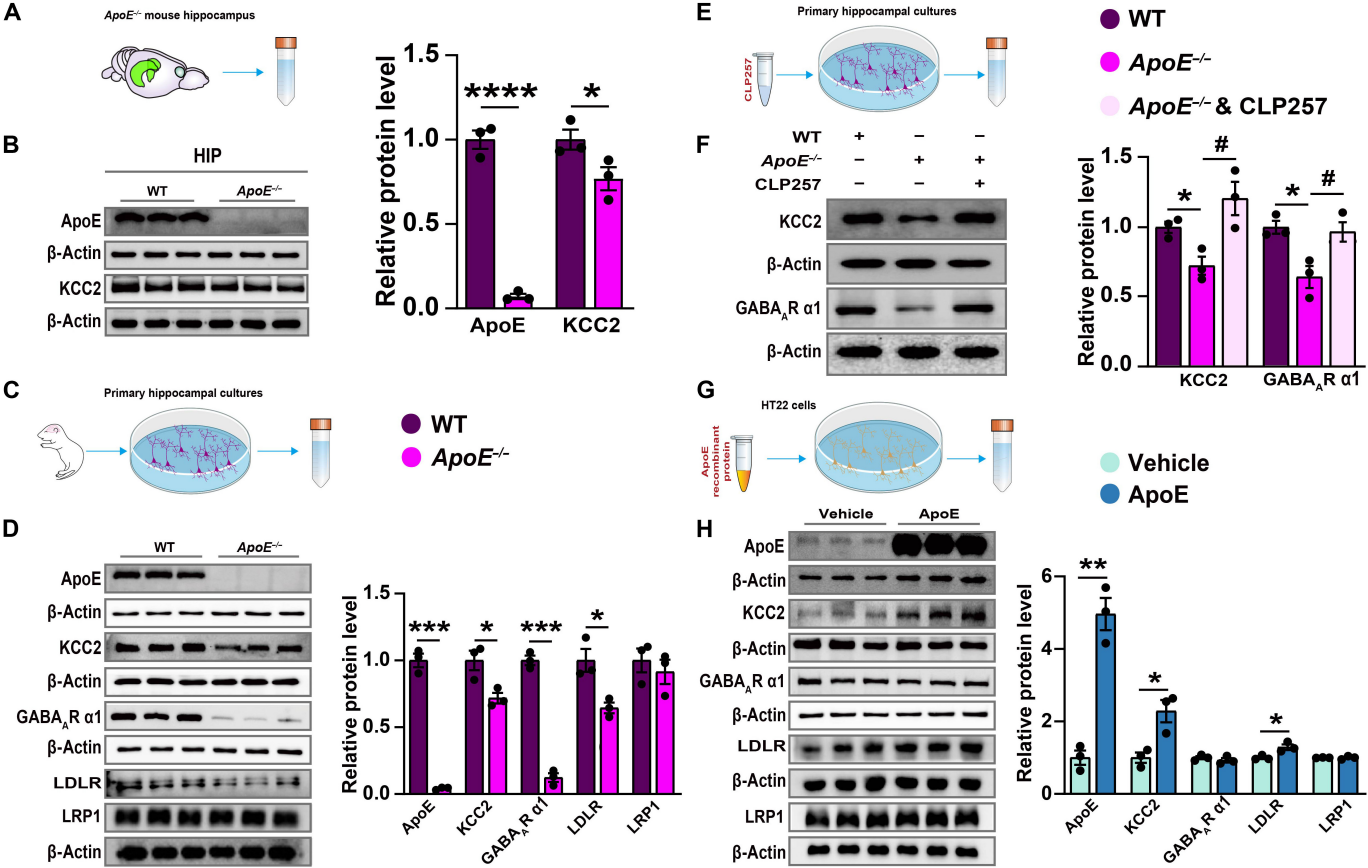
Decreased ApoE receptors, KCC2, and GABA_A_R α1 protein levels in the hippocampus of *ApoE^−/−^* mice. (A) The supernatant obtained from *ApoE^−/−^* mouse hippocampal tissue was collected for subsequent experimental analyses. (B) Representative western blot bands for ApoE and KCC2. Statistical results of these proteins (wild type [WT], *n* = 3; *ApoE^−/−^*, *n* = 3). (C) Primary hippocampal neurons isolated from *ApoE^−/−^* mouse pup brain. (D) Representative western blot bands for ApoE, KCC2, GABA_A_R α1, LDLR, and LRP1. Statistical results of these proteins (WT, *n* = 3; *ApoE^−/−^*, *n* = 3). (E) Primary hippocampal neurons isolated from *ApoE^−/−^* mouse pup brain treated with CLP257. (F) Representative western blot bands for KCC2 and GABA_A_R α1. Statistical results of these proteins (WT, *n* = 3; *ApoE^−/−^*, *n* = 3; *ApoE^−/−^* & CLP257, *n* = 3). (G) Treatment with ApoE recombinant protein in HT22 cells. (H) Representative western blot bands for ApoE, KCC2, GABA_A_R α1, LDLR, and LRP1. Statistical results of these proteins (vehicle, *n* = 3; ApoE, *n* = 3). Values are presented as mean ± SEM. WT vs. *ApoE^−/−^*, *P < 0.05, ***P < 0.001, ****P < 0.0001; *ApoE^−/−^* vs. *ApoE^−/−^* & CLP257, ^#^*P* < 0.05, ^##^*P* < 0.01; vehicle vs. ApoE, *P < 0.05, **P < 0.01. HIP, hippocampus.

Notably, the hippocampus of *ApoE^−/−^* mice showed a significant decrease in KCC2 expression (Fig. 6A and B). Subsequently, we carried out a series of in vitro experiments to dissect the underlying mechanisms by which ApoE regulates KCC2 and GABAergic signaling. Primary hippocampal neurons were isolated from *ApoE^−/−^* mouse pup brain (Fig. 6C). Western blot analysis revealed that compared to wild-type controls, the *ApoE^−/−^* hippocampal cultures exhibited significant reductions in the protein levels of KCC2, GABA_A_R α1, and LDLR (Fig. 6D). Intriguingly, treatment with the KCC2 activator CLP257 (10 μM) for 24 h significantly reversed the decreases in KCC2 and GABA_A_R α1 protein levels (Fig. 6E and F). Moreover, when HT22 cells were treated with ApoE recombinant protein (1 μg/ml) for 24 h (Fig. 6G), there was a significant up-regulation of ApoE, KCC2, and LDLR levels (Fig. 6H). Collectively, these results strongly suggest that ApoE modulates KCC2 expression through its receptors in an in vitro setting.

### The activation of hippocampal GABAergic neurons rescues depression-like behaviors in CSDS-exposed and ApoE-KD mice

**Fig. 7.**
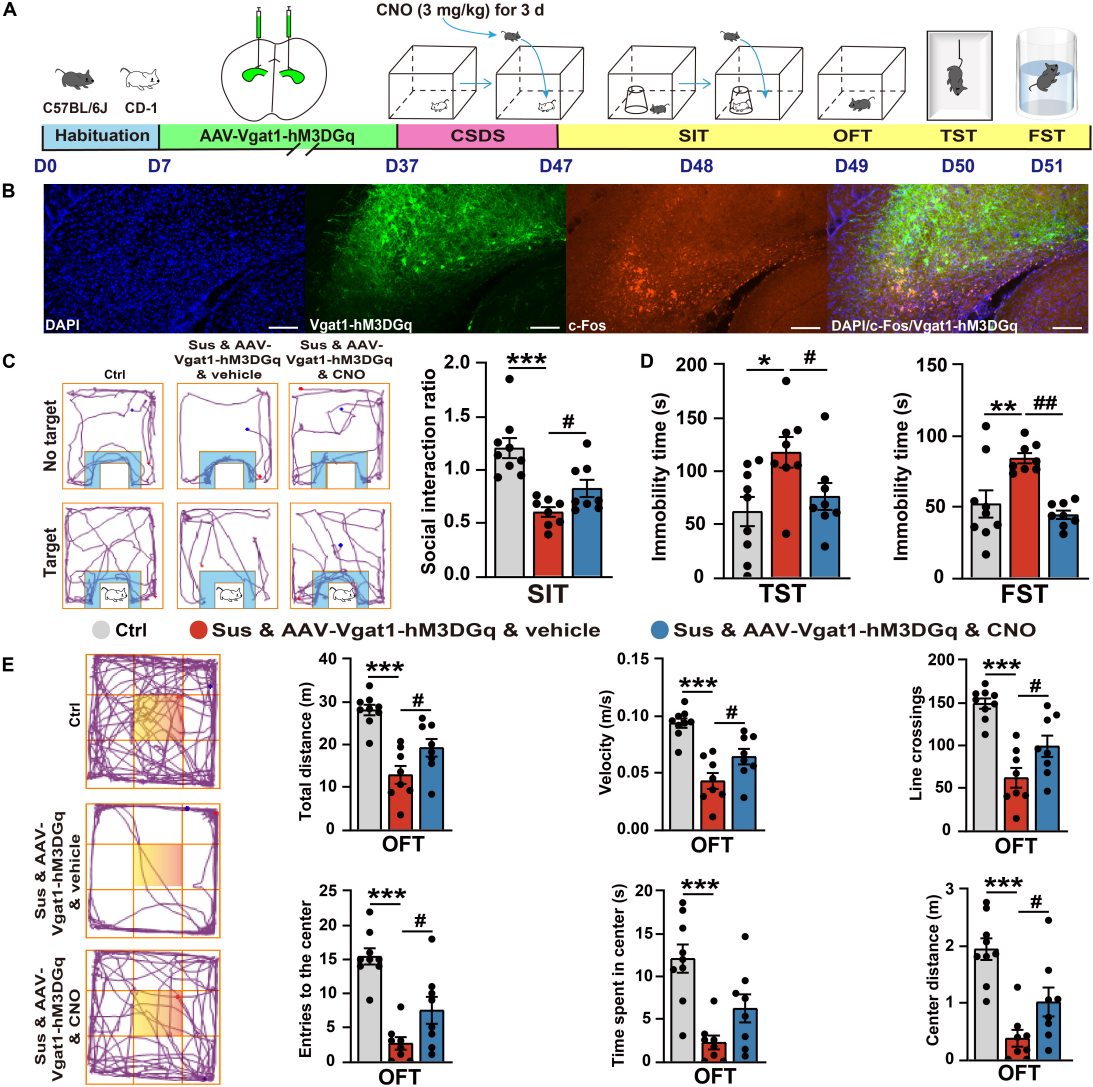
Targeted activation of hippocampal GABAergic neurons reverses depression-like behaviors in mice following CSDS exposure. (A) Experiment flowchart and timeline for habituation, AAV injection, CSDS, clozapine *N*-oxide (CNO) intraperitoneal injection, and behavioral tests (SIT, OFT, TST, and FST). (B) Activation of Vgat1-hM3DGq in hippocampal GABAergic neurons significantly increases c-Fos expression. Scale bar = 100 μm. (C) Representative tracks from the SIT (the blue area is marked as the SI zone). Statistical results of SIR (Ctrl, *n* = 9; Sus & AAV-Vgat1-hM3DGq & vehicle, *n* = 8; Sus & AAV-Vgat1-hM3DGq & CNO, *n* = 8). (D) Statistical results of immobility time in the TST and FST (Ctrl, *n* = 9; Sus & AAV-Vgat1-hM3DGq & vehicle, *n* = 8; Sus & AAV-Vgat1-hM3DGq & CNO, *n* = 8). (E) Representative tracks from the OFT (the yellow area is marked as the central zone). Statistical results of the distance moved, line crossings, speed, entries to the centers, and time/distance spent in the center during the OFT (Ctrl, *n* = 9; Sus & AAV-Vgat1-hM3DGq & vehicle, *n* = 8; Sus & AAV-Vgat1-hM3DGq & CNO, *n* = 8). Values are presented as mean ± SEM. Ctrl vs. Sus & AAV-Vgat1-hM3DGq & vehicle, *P < 0.05, **P < 0.01, ***P < 0.001; Sus & AAV-Vgat1-hM3DGq & vehicle vs. Sus & AAV-Vgat1-hM3DGq & CNO, ^#^*P* < 0.05, ^##^*P* < 0.01.

A diminished GABAergic function has been strongly associated with depression, and this function is regulated by a combination of genetic, developmental, and environmental factors that disrupt the activity of GABAergic interneurons and compromise synaptic integrity [14]. Moreover, in chronic unpredictable mild stress (CUMS) mouse models, there is a notable decrease in the expression levels of GABAergic markers, such as GAD65, in both the hippocampus and mPFC. The restoration of GABAergic function through the use of ketamine or traditional antidepressant medications demonstrates efficacy in reducing depressive symptoms [32]. In our current study, we observed a significant reduction in the protein level of GABA_A_R α1 following exposure to CSDS (Fig. S1A), which suggests an impairment of the GABAergic function. To explore whether the activation of GABAergic neurons could mitigate depression-like behaviors, we employed a chemical genetics approach for neuron-targeted activation. C57BL/6J mice that had been pretreated with AAV-Vgatl-hM3DGq received daily intraperitoneal injections of clozapine *N*-oxide (CNO) at a dosage of 3 mg/kg during the last 3 d of the 10-d CSDS modeling period (Fig. 7A). Furthermore, upon activation with CNO, there was a notable increase in the expression of c-Fos in the CA3 region of the hippocampus (Fig. 7B). When compared to that in the Ctrl group, the SIR in the Sus & AAV-Vgat1-hM3DGq & vehicle group was significantly lower. In contrast, the SIR was significantly elevated in the Sus & AAV-Vgat1-hM3DGq & CNO group after CNO activation (Fig. 7C). Similarly, the immobility times in the TST and FST were significantly longer in the Sus & AAV-Vgat1-hM3DGq & vehicle group relative to those in the Ctrl group. However, these immobility times were significantly reduced in the Sus & AAV-Vgat1-hM3DGq & CNO group following CNO activation (Fig. 7D). Additionally, the Sus & AAV-Vgat1-hM3DGq & CNO group showed a significant improvement in locomotor activity as measured by the OFT after CNO activation (Fig. 7E).

**Fig. 8.**
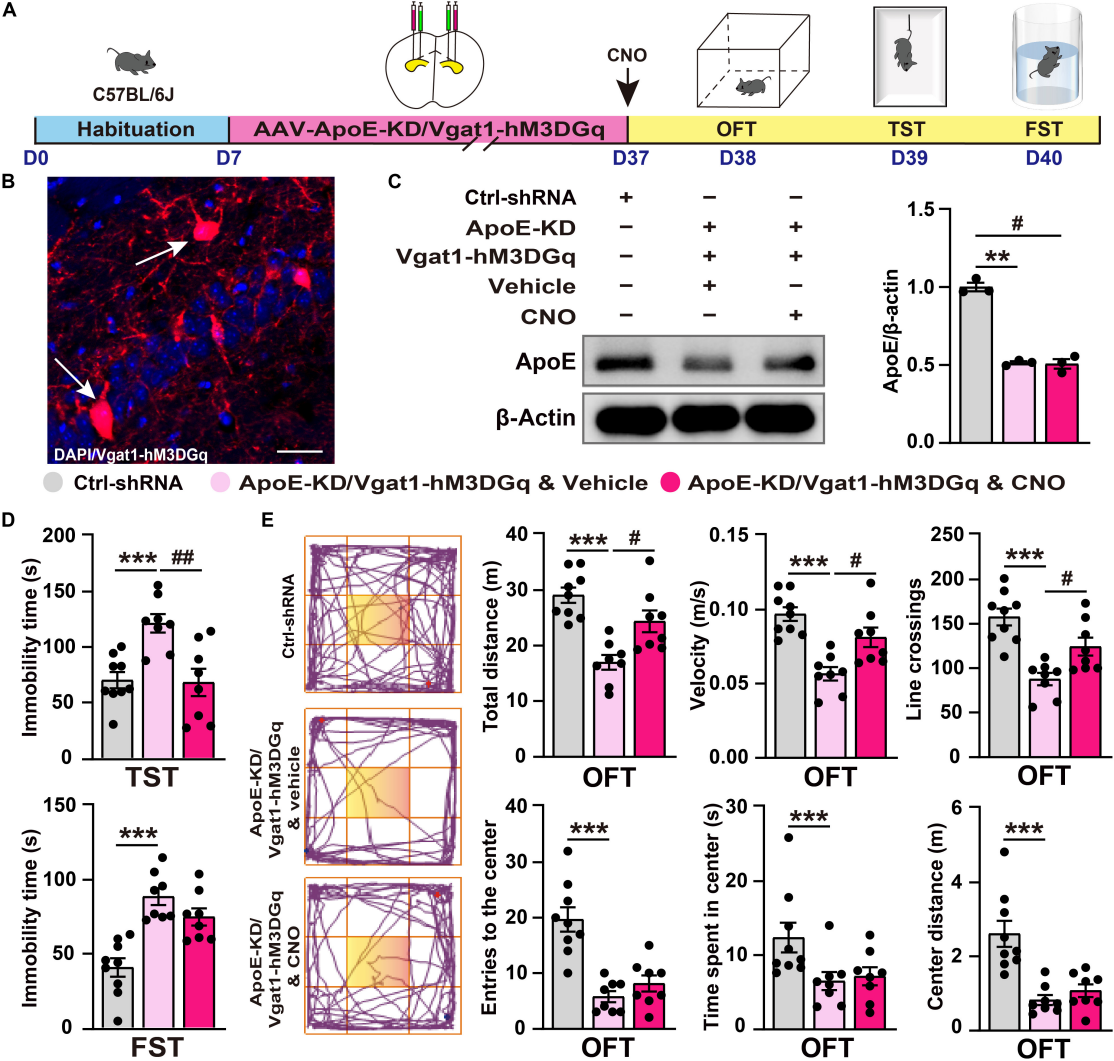
Targeted activation of hippocampal GABAergic neurons partially reverses depression-like behaviors in ApoE-KD mice. (A) Experiment flowchart and timeline for habituation, AAV injection, CNO intraperitoneal injection, and behavioral tests (OFT, TST, and FST). (B) AAV-Vgat1-hM3DGq specifically labels GABAergic neurons in the hippocampus. Scale bar = 20 μm. (C) The protein expression of ApoE confirmed the knockdown effect of AAV-ApoE-KD (Ctrl-shRNA, *n* = 3; ApoE-KD/Vgat1-hM3DGq & vehicle, *n* = 3; ApoE-KD/Vgat1-hM3DGq & CNO, *n* = 3). (D) Statistical results of immobility time in the TST and FST (Ctrl-shRNA, *n* = 9; ApoE-KD/Vgat1-hM3DGq & vehicle, *n* = 8; ApoE-KD/Vgat1-hM3DGq & CNO, *n* = 8). (E) Representative tracks from the OFT (the yellow area is marked as the central zone). Statistical results of the distance moved, line crossings, speed, entries to the centers, and time/distance spent in the center during the OFT (Ctrl-shRNA, *n* = 9; ApoE-KD/Vgat1-hM3DGq & vehicle, *n* = 8; ApoE-KD/Vgat1-hM3DGq & CNO, *n* = 8). Values are presented as mean ± SEM. Ctrl-shRNA vs. ApoE-KD/Vgat1-hM3DGq & vehicle, *P < 0.05, **P < 0.01, ***P < 0.001; ApoE-KD/Vgat1-hM3DGq & vehicle vs. ApoE-KD/Vgat1-hM3DGq & CNO, ^#^*P* < 0.05, ^##^*P* < 0.01.

To elucidate the role of GABAergic synaptic activity in ApoE-KD-induced depression, we coinjected ApoE-KD AAV and Vgat1-hM3DGq AAV into the hippocampus of C57BL/6J mice (Fig. 8A). Immunofluorescence staining confirmed that the Vgat1-hM3DGq AAV selectively labeled GABAergic neurons (Fig. 8B), while western blot analysis detected significantly decreased ApoE protein expression (Fig. 8C). Upon activation with CNO (3 mg/kg), behavioral assays demonstrated that the targeted activation of hippocampal GABAergic neurons partially ameliorated the depression-like behaviors caused by ApoE knockdown. The ApoE-KD/Vgat1-hM3DGq & vehicle group exhibited significantly prolonged immobility time in the TST compared to the Ctrl-shRNA group. However, this increase was significantly reversed in the ApoE-KD/Vgat1-hM3DGq & CNO group following CNO activation (Fig. 8D). Similarly, the ApoE-KD/Vgat1-hM3DGq & CNO group showed a substantial improvement in locomotor activity as measured by the OFT after CNO treatment (Fig. 8E). Together, these results demonstrate that the targeted activation of hippocampal GABAergic neurons can partially reverse the depression-like behaviors induced by both CSDS exposure and ApoE-KD, highlighting the critical role of GABAergic synaptic activity in the pathological process of ApoE-related depression.

### CLP290 ameliorates depression-like behaviors induced by CSDS exposure and ApoE-KD

**Fig. 9.**
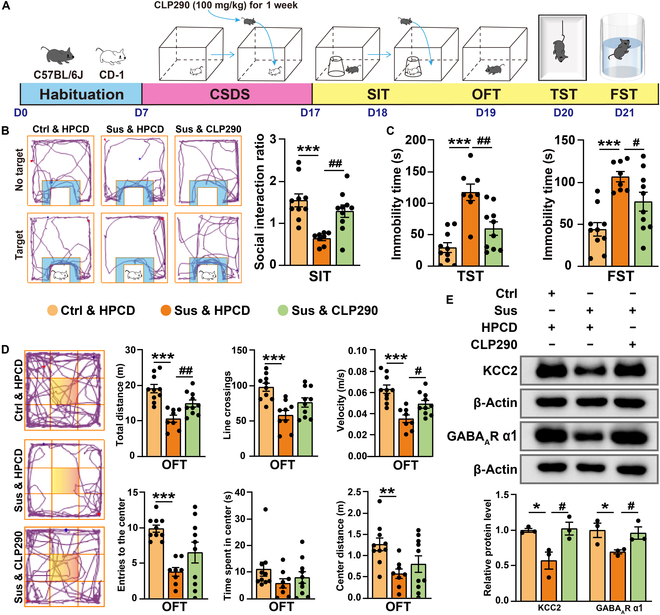
CLP290 reverses depression-like behaviors induced by CSDS exposure. (A) Experiment flowchart and timeline for habituation, CSDS, CLP290 intraperitoneal injection, and behavioral tests (SIT, OFT, TST, and FST). (B) Representative tracks from the SIT (the blue area is marked as the SI zone). Statistical results of SIR (Ctrl & HPCD, *n* = 10; Sus & HPCD, *n* = 8; Sus & CLP290, *n* = 10). (C) Statistical results of immobility time in the TST and FST (Ctrl & HPCD, *n* = 10; Sus & HPCD, *n* = 8; Sus & CLP290, *n* = 10). (D) Representative tracks from the OFT (the yellow area is marked as the central zone). Statistical results of the distance moved, line crossings, speed, entries to the centers, and time/distance spent in the center during the OFT (Ctrl & HPCD, *n* = 10; Sus & HPCD, *n* = 8; Sus & CLP290, *n* = 10). (E) Representative western blot bands for KCC2 and GABA_A_R α1 in the hippocampus. Statistical results of KCC2 and GABA_A_R α1 protein levels in the hippocampus (Ctrl & HPCD, *n* = 3; Sus & HPCD, *n* = 3; Sus & CLP290, *n* = 3). Values are presented as mean ± SEM. Ctrl & HPCD vs. Sus & HPCD, *P < 0.05, **P < 0.01, ***P < 0.001; Sus & HPCD vs. Sus & CLP290, ^#^*P* < 0.05, ^##^*P* < 0.01. HPCD, hydroxypropyl-β-cyclodextrin.

Previous studies have shown that the protein levels of KCC2 and GABA_A_R α1 are significantly reduced in the hippocampus of mice following exposure to CSDS (Fig. S1A). To examine the role of KCC2 in depression, we administered another KCC2 activator, CLP290, to mice that had been exposed to CSDS. The mice received continuous intraperitoneal injections of CLP290 (100 mg/kg) for 7 consecutive days during the CSDS modeling process. In contrast, control mice received equivalent volumes of the hydroxypropyl-β-cyclodextrin (HPCD) solvent (Fig. 9A). Behavioral tests revealed that treatment with CLP290 significantly ameliorated depression-like behaviors. The SIR was significantly reduced in the Sus & HPCD group relative to that in the Ctrl & HPCD group, while it was significantly elevated in the Sus & CLP290 group (Fig. 9B). In contrast to the Ctrl & HPCD group, the Sus & HPCD group displayed a significant rise in immobility duration during both the TST and FST. On the other hand, the Sus & CLP290 group exhibited a significant decline in immobility time compared to the Sus & HPCD group (Fig. 9C). Furthermore, the locomotor activity in the OFT was substantially higher in the Sus & CLP290 group than in the Sus & HPCD group (Fig. 9D). According to western blot data, CLP290 administration led to a significant up-regulation of KCC2 and GABA_A_R α1 expression levels in the Sus & CLP290 group as compared to those in the Sus & HPCD group (Fig. 9E).

**Fig. 10.**
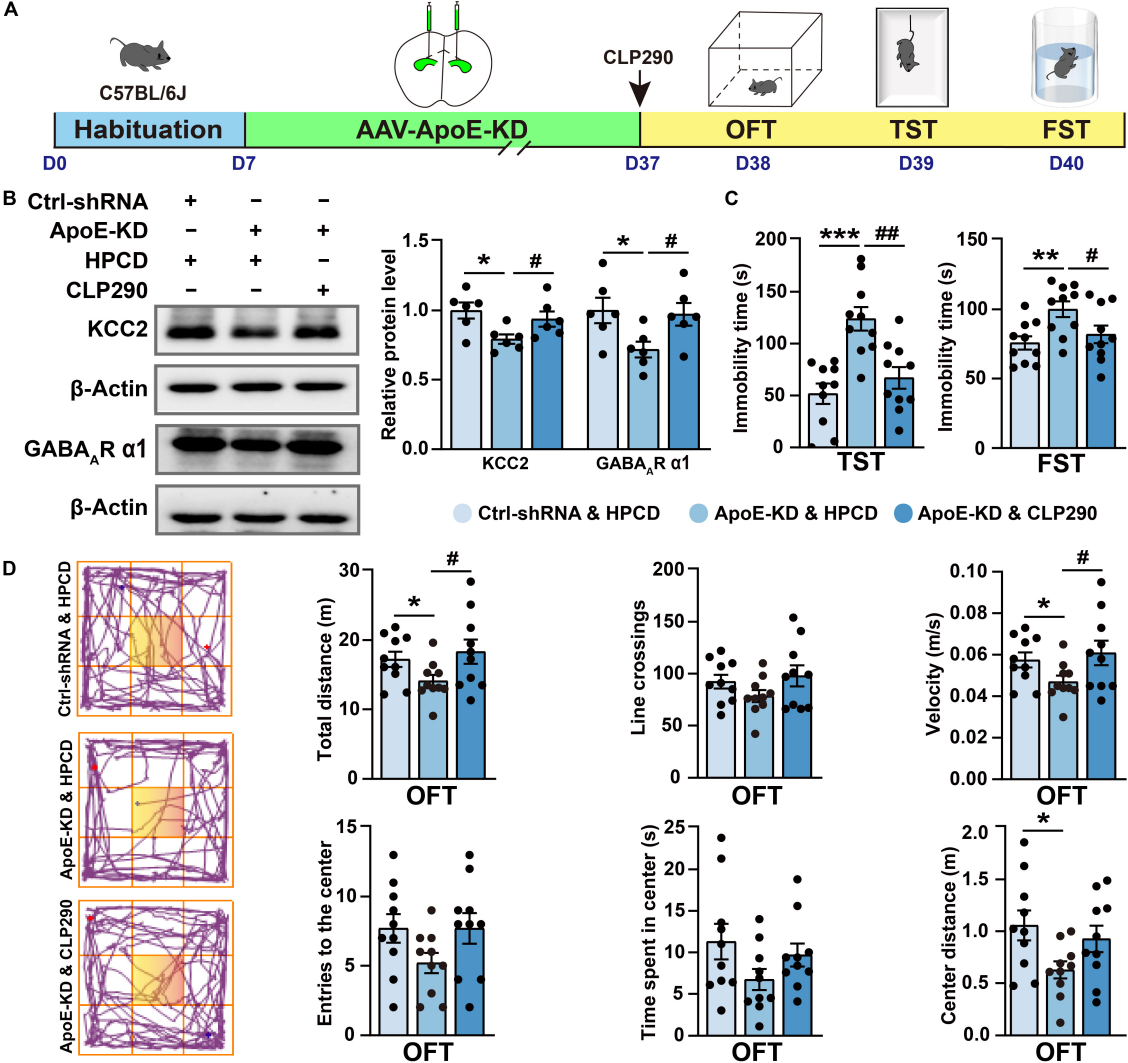
CLP290 improves depression-like behaviors induced by ApoE-KD. (A) Experiment flowchart and timeline for habituation, AAV injection, CLP290 intraperitoneal injection, and behavioral tests (OFT, TST, and FST). (B) Representative western blot bands for KCC2 and GABA_A_R α1 in the hippocampus. Statistical results of KCC2 and GABA_A_R α1 protein levels in the hippocampus (Ctrl-shRNA & HPCD, *n* = 3; ApoE-KD & HPCD, *n* = 3; ApoE-KD & CLP290, *n* = 3). (C) Statistical results of immobility time in the TST and FST (Ctrl-shRNA & HPCD, *n* = 10; ApoE-KD & HPCD, *n* = 10; ApoE-KD & CLP290, *n* = 10). (D) Representative tracks from the OFT (the yellow area is marked as the central zone). Statistical results of the distance moved, line crossings, speed, entries to the centers, and time/distance spent in the center during the OFT (Ctrl-shRNA & HPCD, *n* = 10; ApoE-KD & HPCD, *n* = 10; ApoE-KD & CLP290, *n* = 10). Values are presented as mean ± SEM. Ctrl-shRNA & HPCD vs. ApoE-KD & HPCD, *P <0.05, **P <0.01, ***P <0.001; ApoE-KD & HPCD vs. ApoE-KD & CLP290, ^#^*P* < 0.05, ^##^*P* < 0.01.

Next, we investigated the impact of CLP290 on depression-like behaviors in ApoE-KD mice (Fig. 10A). After 7 d of intraperitoneal injection treatment with CLP290 (100 mg/kg), behavioral tests demonstrated a significant improvement in depression-like behaviors in ApoE-KD mice. The immobility time in the TST and FST was significantly longer in the ApoE-KD & HPCD group than in the Ctrl-shRNA & HPCD group, but it was significantly shorter in the ApoE-KD & CLP290 group (Fig. 10C). The ApoE-KD & CLP290 group also exhibited a significantly enhanced locomotor activity in the OFT when compared to the ApoE-KD & HPCD group (Fig. 10D). Moreover, the western blot results demonstrated that the protein expression levels of KCC2 and GABA_A_R α1 were significantly reduced in the ApoE-KD & HPCD group as compared to those in the Ctrl-shRNA & HPCD group. However, this reduction was significantly reversed after the administration of CLP290 treatment (Fig. 10B). Altogether, these findings suggest that KCC2 plays a pivotal role in CSDS-induced depression. ApoE may be involved in the pathophysiology of depression by regulating KCC2-mediated GABAergic synaptic function.

## Discussion

**Fig. 11.**
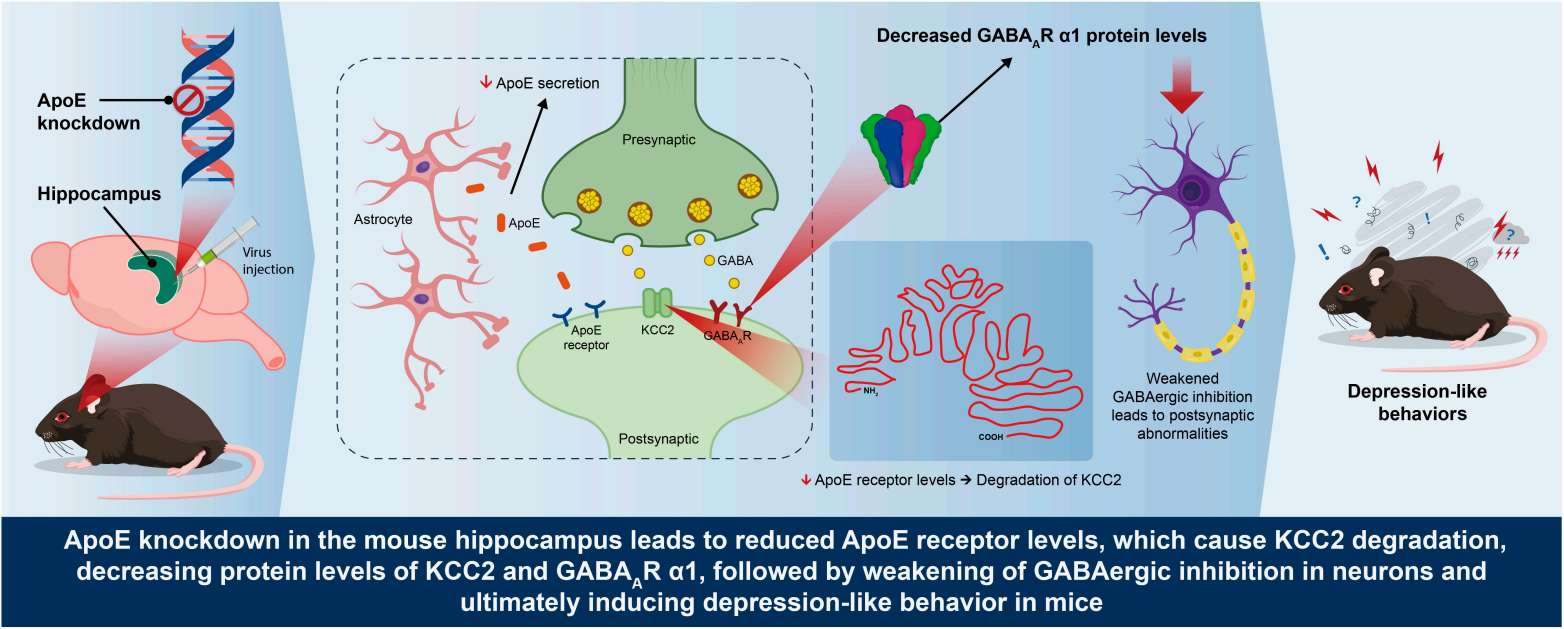
A working model illustrating the mechanism by which ApoE regulates GABA_A_R-mediated inhibition and depression-like behaviors in mice. ApoE deficiency leads to reduced activity of the ApoE receptors’ pathway on neurons, which promotes KCC2 degradation. This results in decreased protein levels of KCC2 and GABA_A_R α1, weakening GABAergic inhibition in neurons and ultimately inducing depression-like behaviors in mice.

Our study demonstrates that ApoE plays a critical role in modulating GABAergic signaling and contributes to the pathophysiology of depression by regulating the expression and function of KCC2. This conclusion is supported by multiple lines of evidence: (a) ApoE-KD in the hippocampus decreased the expression of GABA_A_R α1 and the amplitude of mIPSC in CA1 pyramidal neurons, indicating impaired GABAergic synaptic function. (b) ApoE-KD significantly reduced the level of KCC2, resulting in insufficient GABAergic inhibition. Conversely, treatment with KCC2 activators, CLP290 and CLP257, restored GABAergic synaptic function. (c) Overexpression of ApoE in the hippocampus, targeted activation of GABAergic neurons, and restoration of KCC2 function rescued depression-like behaviors in mice. Our findings underscore the roles of ApoE and KCC2 in regulating GABAergic synaptic activity and influencing depression-like behaviors in mice (Fig. 11). These insights offer potential therapeutic targets for clinical intervention.

The hippocampus, mPFC, and NAc are key interconnected brain regions implicated in the pathophysiology of depression [33]. As a brain region highly sensitive to stress, the hippocampus exhibited pronounced early pathological changes in both the CSDS and CUMS mouse models [16,27]. Notably, studies have demonstrated that ApoE promotes memory consolidation by enriching acetylated histones in promoter regions and enhancing the transcription of immediate-early genes. Moreover, specific knockdown of ApoE in hippocampal astrocytes induces notable learning and memory deficits [11]. Although dysfunction of the mPFC is associated with mental disorders such as depression and anxiety, CSDS may exacerbate anxiety- and depression-like behaviors by inducing synaptic structural damage and mitochondrial dysfunction in the mPFC [34–36]. ApoE4 impairs mitochondrial structure and function, thereby leading to oxidative stress and subsequent alterations in synaptic plasticity and cognitive performance [37]. CSDS can substantially up-regulate the expression of matrix metalloproteinase-8 in peripheral immune cells. This up-regulation then affects the normal function of neurons by altering the structure of the extracellular space in the NAc [38]. Notably, ApoE4 derived from astrocytes substantially up-regulates the expression of matrix metalloproteinase-9. This up-regulation disrupts tight-junction proteins and, consequently, impairs the integrity of the blood–brain barrier [39,40]. Our results indicate that in the CSDS model, the number of astrocytes and the levels of ApoE in the hippocampus were significantly reduced. In contrast, no changes in ApoE levels were observed in the mPFC and NAc brain regions. Thus, we propose that the depression-like behaviors following CSDS exposure are mainly attributable to ApoE deficiency in the hippocampus. We hypothesize that ApoE may also regulate the function of the mPFC and NAc in CSDS-induced depression-like behaviors via distinct pathways.

The dysregulation of astrocytic function has been associated with depression, which is characterized by synaptic dysfunction, synapse loss, and neuronal death [41,42]. In the brain of healthy adults, ApoE is mainly synthesized and secreted by astrocytes, accounting for approximately 90% of its total expression. Notably, neurons can also release ApoE under stress and pathological conditions [6]. Elevated ApoE levels have been shown to enhance debris clearance, suppress inflammatory responses, and facilitate the delivery of lipids to neurons, thereby bolstering neuronal resilience [43]. In our study, we discovered that CSDS can significantly decrease the expression level of ApoE in the hippocampus. To further elucidate its cell-specific mechanism, in subsequent studies, we will employ a hippocampal astrocyte conditional ApoE knockout mouse model to investigate the regulatory role of astrocyte-derived ApoE in the pathophysiology of depression. In humans, ApoE has 3 major allele variants: ε2 (ApoE2), ε3 (ApoE3), and ε4 (ApoE4). However, in animal models, there are no subtypes of ApoE, and its functional characteristics are analogous to those of human ApoE3 [44–46]. The ApoE4 protein is less stable than the ApoE3 protein and has a higher propensity to be hydrolyzed into neurotoxic protein fragments. These neurotoxic substances derived from ApoE4 ultimately lead to synaptic dysfunction by decreasing the supply of synaptic lipids and reducing the density of hippocampal dendritic spines [24,47]. Our study primarily focuses on the physiological function of ApoE, without making a distinction between ApoE isoforms. Further research is required to elucidate the roles of different ApoE isoforms, such as ApoE4 versus ApoE2, in the regulation of GABAergic synaptic function and in the context of depression.

The primary function of KCC2 is to maintain intracellular Cl^−^ concentration by extruding K^+^ and Cl^−^ from the cell, thereby regulating GABA_A_R-mediated inhibitory synaptic transmission. Recent studies have highlighted the roles of KCC2 in the CNS and its association with neuropathological conditions, including Alzheimer’s disease (AD) [31,48–53]. Chloride dysregulation in AD is associated with cognitive decline. Specifically, the loss of KCC2 gives rise to ionic imbalance and neuronal hyperexcitability and ultimately results in neuronal death. Preventing the loss of KCC2 might potentially slow down or even reverse certain symptoms of AD [54]. Dysfunction of KCC2 disrupts neuronal chloride homeostasis, impairing the formation of the Cl^−^ gradient and consequently leading to an imbalance in neural network E/I [55]. In the animal model of treatment-resistant depression, the expression level of KCC2 in the lateral habenular nucleus is diminished. This observation suggests that the modulation of KCC2 could potentially serve as a viable target for the treatment of treatment-resistant depression. Cumulatively, the aforementioned studies imply that KCC2 may be implicated in the pathophysiology of certain types of depression [56,57]. However, its specific regulatory mechanism remains unexplored, particularly in the context of stress-induced depression-like behaviors. In our study, we employed mice exposed to CSDS and detected a significant reduction in KCC2 levels. The knockdown of ApoE significantly decreased the expression of both KCC2 and GABA_A_R α1. Conversely, treatment with the KCC2 activator, CLP290, significantly alleviated the depression-like behaviors in mice induced by CSDS exposure and ApoE-KD. These findings indicate that KCC2 plays a pivotal role in the development of depression, thereby offering a crucial theoretical foundation for the formulation of novel antidepressant strategies that target KCC2.

As is well-known, ApoE4 is a prevalent genetic risk factor for both AD and depression. Clinical investigations suggest that the ApoE4 homozygous genotype is linked to a notably elevated risk of AD, and ApoE4 carriers encounter a 20% higher likelihood of experiencing depressive symptoms compared to noncarriers [44,46,58–63]. Furthermore, patients with major depression frequently exhibit mild cognitive impairment and show a markedly elevated risk of developing AD [64–67]. Current evidence indicates that ApoE and KCC2 serve critical regulatory functions in the pathology of both AD and depression. We hypothesize that the ApoE–KCC2 signaling pathway-mediated GABAergic synaptic dysfunction may play a pivotal role in the common pathophysiology of these disorders. Previous studies have shown that ApoE can regulate downstream synaptic activity through neuronal receptor pathways [8,9]. Our findings indicate that the expression levels of these receptors also significantly decrease in the absence of ApoE. Interestingly, both ApoE receptors (LDLR and LRP1) and KCC2 are enriched on the neuronal membrane, where they interact directly. This interaction likely prevents KCC2 phosphorylation and ubiquitination, reducing its degradation and supporting its stability [31]. These results imply that ApoE receptors confer stability to KCC2 on the neuronal cell membrane via protein–protein interactions, which sustain appropriate levels of KCC2 and contribute to the stabilization of GABA_A_R-mediated inhibition. These findings shed light on the potential mechanism through which ApoE regulates the expression of KCC2.

GABAergic synaptic dysfunction is a common pathological feature of various neuropsychiatric diseases. In patients with schizophrenia, GABAergic synaptic dysfunction indirectly affects the function of the dopamine system by regulating glutamatergic signaling pathways, leading to typical positive symptoms alongside negative symptoms [68]. Furthermore, GABAergic synaptic dysfunction is one of the key pathological mechanisms in AD, characterized by impaired signaling of the inhibitory neurotransmitter GABA. This dysfunction leads to an imbalance between neuronal E/I, resulting in abnormal neural network oscillations and impaired synaptic plasticity, ultimately accelerating neurodegeneration and cognitive decline in AD patients [47,69]. Consistent with our study, previous research has demonstrated that synaptic dysfunction is a hallmark of depression, and interventions targeting the GABAergic system have been shown to alleviate depressive symptoms [70]. Moreover, the neurotoxic properties of ApoE4 have been linked to the impairment of GABAergic interneurons and the disruption of hippocampal neurogenesis in murine models [24]. Additionally, astrocyte-specific ApoE knockdown in the hippocampus has been associated with notable reductions in dendritic spine density [11]. The attenuation of GABAergic inhibitory neurotransmission leading to E/I imbalance may serve as a common pathological basis for these neuropsychiatric disorders. In our research, following the use of AAV to specifically knock down ApoE in the hippocampus, RNA-seq analysis disclosed that numerous synaptic-related genes were down-regulated. The patch-clamp results demonstrated that the amplitude of mIPSC in hippocampal CA1 pyramidal neurons was significantly reduced, which suggests the presence of postsynaptic abnormalities. The targeted activation of hippocampal GABAergic neurons significantly alleviated depression-like behaviors. These findings imply that ApoE is involved in depression-like behaviors by modulating GABAergic synaptic function. Given the substantial heterogeneity of GABAergic neurons, future studies are required to elucidate the roles of specific subtypes, such as SST+ and PV+ neurons, in the context of depression [71].

In conclusion, we delved into the underlying mechanisms by which ApoE influences depression-like behaviors. Exposure to CSDS or the presence of ApoE deficiency can give rise to a decrease in KCC2 expression within the hippocampus. This decrease subsequently leads to a disruption in GABAergic synaptic function and contributes to the manifestation of depression-like behaviors in mice. Treatment with the KCC2 activator, CLP290, effectively restored KCC2 levels, enhanced GABAergic function, and mitigated depression-like behaviors. These findings underscore the pivotal roles of ApoE and KCC2 in the pathological mechanism of depression, offering valuable perspectives for future clinical research and the formulation of potential therapeutic approaches.

## Methods

### Animals

Eight- to 9-month-old retired male CD-1 mice, 8-week-old adult male *ApoE^−/−^* mice, and C57BL/6J mice were purchased from Jiangsu GemPharmatech Co., Ltd. All experimental mice were maintained in a specific-pathogen-free environment, with provision for tap water and regular feed. The research protocols were reviewed and approved by the Experimental Animal Ethics Committee of Anhui Medical University under approval number LLSC20230324.

### Viral injection

Viral injections were administered to the hippocampus using the following coordinates in relation to the bregma after the dura mater was exposed: dorsoventral −1.9 mm, anteroposterior −2.00 mm, and mediolateral ±1.5 mm. The hippocampal region was bilaterally injected with 1.0 μl of the AAV vector using a microinjector (Legato 130, KD Scientific, USA). One month later, the effect of virus transfection was verified. The AAV sequence is as follows: (a) Vgat1 neuron-targeted activation AAV: rAAV-Vgat1-hM3DGq-EGFP, (b) ApoE-OE AAV: rAAV-EF1α-ApoE-P2A-EGFP-WPRE-hGH polyA, (c) ApoE-KD AAV: rAAV-ApoE-RNAi-EGFP.

### Drugs

#### Clozapine *N*-oxide

CNO acts on cells expressing hM3DGq to depolarize and enhance cell excitability. CNO was dissolved in physiological saline and given to mice via intraperitoneal injection (3 mg/kg) after they were pretreated with AAV-Vgat1-hM3DGq.

#### CLP290

CLP290 is an oral activator of KCC2 [31]. Daily intraperitoneal injections of CLP290 (100 mg/kg, dissolved in HPCD solution) were administered for 1 week. At the same time, the HPCD solvent control group was set up.

#### CLP257

CLP257 is a selective KCC2 activator that specifically enhances K^+^–Cl^−^ cotransport and restores chloride transport in neurons with impaired KCC2 activity [31]. Primary hippocampal neurons from *ApoE^−/−^* mice were treated with CLP257 (10 μM) or dimethyl sulfoxide control solution for 24 h.

#### ApoE recombinant protein

The mouse ApoE recombinant protein (HEK293, His) is expressed in HEK293 cells with a His tag at the N-terminus. HT22 cells were treated with ApoE recombinant protein (1 μg/ml) or dimethyl sulfoxide control solution for 24 h.

### Chronic social defeat stress

All CSDS experiments were performed according to a standard protocol [28]. Retired male CD-1 mice were tested for aggressive and territorial tendencies at the beginning of the trial. The latency to attack was measured for 1 min after a nonexperimental C57BL/6J mouse was placed in the open cage of a CD-1 mouse that was kept separately. This screening was conducted once daily for 3 consecutive days. CD-1 aggressors were defined as CD-1 mice that launched an assault within 1 min. Over the course of 10 d, each C57BL/6J mouse was exposed to a distinct CD-1 aggressor for 10 min every day. The C57BL/6J mice were kept apart from the aggressor for 24 h after each interaction, with a perforated wall separating them. Control mice were housed under similar conditions but were not subjected to aggression.

### Behavioral testing

#### Social interaction test

The SIT is employed to simulate social activities in a social environment, observing experimental mice’s interaction with unfamiliar targets to evaluate the presence or absence of social avoidance behavior. The experimental setup included a transparent isolation room (10 × 6 cm, perforated) containing a CD-1 mouse centrally positioned along one side of an open field (45 × 45 cm). A rectangular zone (15 × 25 cm) around the isolation chamber was marked as the social interaction (SI) zone. The test comprised 2 phases: (a) No target: For 2.5 min, the experimental mice were allowed to roam freely in the center of the open field. The total time spent in the SI zone was recorded. (b) Target: A novel CD-1 mouse was placed in the isolation room. The experimental mice were placed in the center of the arena for 2.5 min, and the total amount of time spent in the SI zone was recorded. SIR = (time spent in the SI zone with target/time spent in the SI zone with no target). In the CSDS model, we utilize the SIR to classify experimental mice into behavioral phenotypes. An SIR >1 indicates a Res phenotype, whereas an SIR <1 identifies a Sus phenotype [28,29]. Unless otherwise stated, all subsequent experiments in the CSDS group were conducted using the Sus phenotype mice.

#### Tail suspension test

To ensure that each mouse’s head stayed approximately 10 cm off the ground, each mouse’s tail (about 1 cm from the tip) was taped securely and hung from a tail suspension device for the TST. The test duration was 6 min, with immobility time during the final 4 min used for statistical analysis. The mice were considered immobile if they remained absolutely still. The method of tape application was standardized across all trials to ensure consistency.

#### Forced swimming test

FST was conducted as previously described [72]. The test duration was 6 min, with immobility time during the final 4 min used for statistical analysis. Immobility was defined as floating with just enough motion to maintain the head above the water’s surface.

#### Open-field test

The open field (45 × 45 cm) was divided into 9 squares (15 × 15 cm). The center square was defined as the central zone. The amount of distance moved, line crossings (the number of times the mice crossed the square in the open field), speed, center entries (the number of times mice entered the central zone of the open field), time spent in the center, and distance spent in the center were recorded throughout the 5-min observation period. The open field was thoroughly cleaned with 75% ethanol after each session to avoid olfactory influence.

#### Sucrose preference test

Over 5 d, the SPT was conducted. For the first 3 d, the mice had access to both a 1% sucrose solution and regular water, with bottle positions alternated every 6 h to avoid side preference. The last 2 d involved 18 h of food and water deprivation, followed by a 6-h drinking test with preweighed bottles. During this phase, bottle positions were changed every 2 h. Sucrose preference rate (%) = [sucrose intake/(sucrose intake + water intake)] × 100%.

### Western blotting

Western blot was conducted as previously described [72]. The primary antibody including anti-GFAP (Servicebio, GB12100-100), anti-ApoE (CST, 49285S), anti-GABA_A_R α1 (Abcam, ab94585), anti-KCC2 (CST, 94725S), anti-NMDAR2B (Proteintech, 21920), anti-GluR2 (Proteintech, 11994-1-AP), anti-PSD95 (Abcam, ab18258), anti-LDLR (Proteintech, 66414-1), anti-LRP1 (IPODIX, IPD-ANM3444), and anti-β-actin (Affinity, AF7081). We followed the manufacturer’s recommended antibody dilution ratios.

### Immunofluorescence staining

Immunofluorescence staining was conducted as previously described [72]. The primary antibody was against ApoE (CST, 49285S), GFAP (Servicebio, GB12096), NeuN (Sigma, MAB377X), Iba1 (Servicebio, GB15105), KCC2 (CST, 94725S), or LDLR (Proteintech, 66414-1). We followed the manufacturer’s recommended antibody dilution ratios.

### Enzyme-linked immunosorbent assay

The supernatant was collected after centrifugation of hippocampal tissue lysates for subsequent experiments. ELISA kits were used to quantify Glu and GABA levels (ELK Biotechnology, ELK8591/ELK8234).

### Quantitative real-time polymerase chain reaction

qPCR was conducted as previously described [72]. Standard manufacturer protocols were followed for RNA extraction and complementary DNA synthesis. qPCR amplification was carried out using SYBR Green Pro Taq HS Premix with the following primer sets:

• [GFAP]-Forward, 5′-CCCTGGCTCGTGTGGATTT-3′
• [GFAP]-Reverse, 5′-GACCGATACCACTCCTCTGTC-3′
• [ApoE]-Forward, 5′-CTCCCAAGTCACACAAGAACTG-3′
• [ApoE]-Reverse, 5′-CCAGCTCCTTTTTGTAAGCCTTT-3′
• [KCC2]-Forward, 5′-GGGCAGAGAGTACGATGGC-3′
• [KCC2]-Reverse, 5′-CCCTGGGGTAGGTTGGTGTA-3′

### Whole-cell patch-clamp recording

Hippocampal brain slices with a thickness of 350 μm were prepared by a vibrating slicer in the cutting solution of an ice–water mixture. The brain slices were transferred to 37 °C artificial cerebrospinal fluid for recovery for 30 min and then placed in recording solution (containing 1 μM tetrodotoxin). The recording pipettes (impedance 3 to 5 MΩ) were filled with the internal solution. Whole-cell recording was performed under a visual patch-clamp system (neurons were voltage-clamped at −70 mV for mEPSC or 0 mV for mIPSC, with recordings sampled at 20 kHz and low-pass filtered at 1 kHz), and offline analysis was performed using Clampfit 10.7 (Molecular Devices). All solutions were oxygenated with 95% O_2_ and 5% CO_2_ mixed gases. The cutting solution and recording solution compositions were prepared as previously described [72].

### RNA-seq and protein–protein interaction network analyses

RNA-seq and protein–protein interaction network analyses was conducted as previously described [72]. After 1 month of stable AAV expression, hippocampal tissues from Ctrl-shRNA and ApoE-KD mice were collected for analysis. Differentially expressed genes (DEGs) relevant to our research focus were identified through enrichment analysis conducted by Shanghai Ou Yi Biomedical Technology Co., Ltd. The DESeq R software (version 1.38.2) was used to perform differential expression analysis between the Ctrl-shRNA and ApoE-KD groups (*n* = 4 per group). DEGs were considered statistically significant if their *P* < 0.05. The clusterProfiler R package (version 4.6.0) was used to conduct Gene Ontology and Kyoto Encyclopedia of Genes and Genomes pathway enrichment studies on DEGs.

### Molecular docking

The 3-dimensional structures of LDLR and KCC2 were obtained from the Protein Data Bank (https://www.rcsb.org/). Molecular docking simulations were carried out using the HDOCK web server (http://hdock.phys.hust.edu.cn/), and the resulting complexes were analyzed and visualized using PyMOL 3.0.

### Cell processing

HT22 cells or primary hippocampal neurons isolated from *ApoE^−/−^* mouse pups were plated in 12-well plates at a uniform density of 3 × 10^5^ cells/well. Primary hippocampal neurons received 10 μM CLP257 or solvent control, while HT22 cells were treated with 1 μg/ml ApoE or solvent control. All cells were maintained at 37 °C with 5% CO_2_ for 24 h.

### Coimmunoprecipitation

The hippocampal tissue homogenate was incubated with 2 μg of anti-KCC2 antibody (CST, 94725S) and 500 μg of total protein in immunoprecipitation buffer at 4 °C for 12 h, followed by the addition of 20 μl of Pierce Protein A Agarose (Thermo, 20333) and rotation overnight at 4 °C. After stringent washing (with protease inhibitors), the immunocomplexes were eluted using an acidic elution buffer. Subsequently, immunoblotting analysis was performed.

### Statistical analysis

Statistical significance between groups was assessed using an unpaired Student *t* test, one-way analysis of variance (ANOVA), or 2-way ANOVA, as specified in the figure legends. All data are expressed as mean ± standard error of the mean, and *P* < 0.05 indicates a statistically significant result.

## Data Availability

The full dataset is provided in the main text and Supplementary Materials. Researchers may also obtain extra data pertinent to this study by contacting the corresponding authors.
