# Supplementary material for: Targeting ApoE–KCC2 Signaling Rescues GABAergic Synaptic Dysfunction and Depression-like Behaviors in Mice
Source: Research (Wash D C). 2025 Jun 17;8:0746. doi: 10.34133/research.0746 (PMC12173456; doi:10.34133/research.0746)
Supplement: Supplementary 1 — Figs. S1 and S2 [file research.0746.f1.docx]

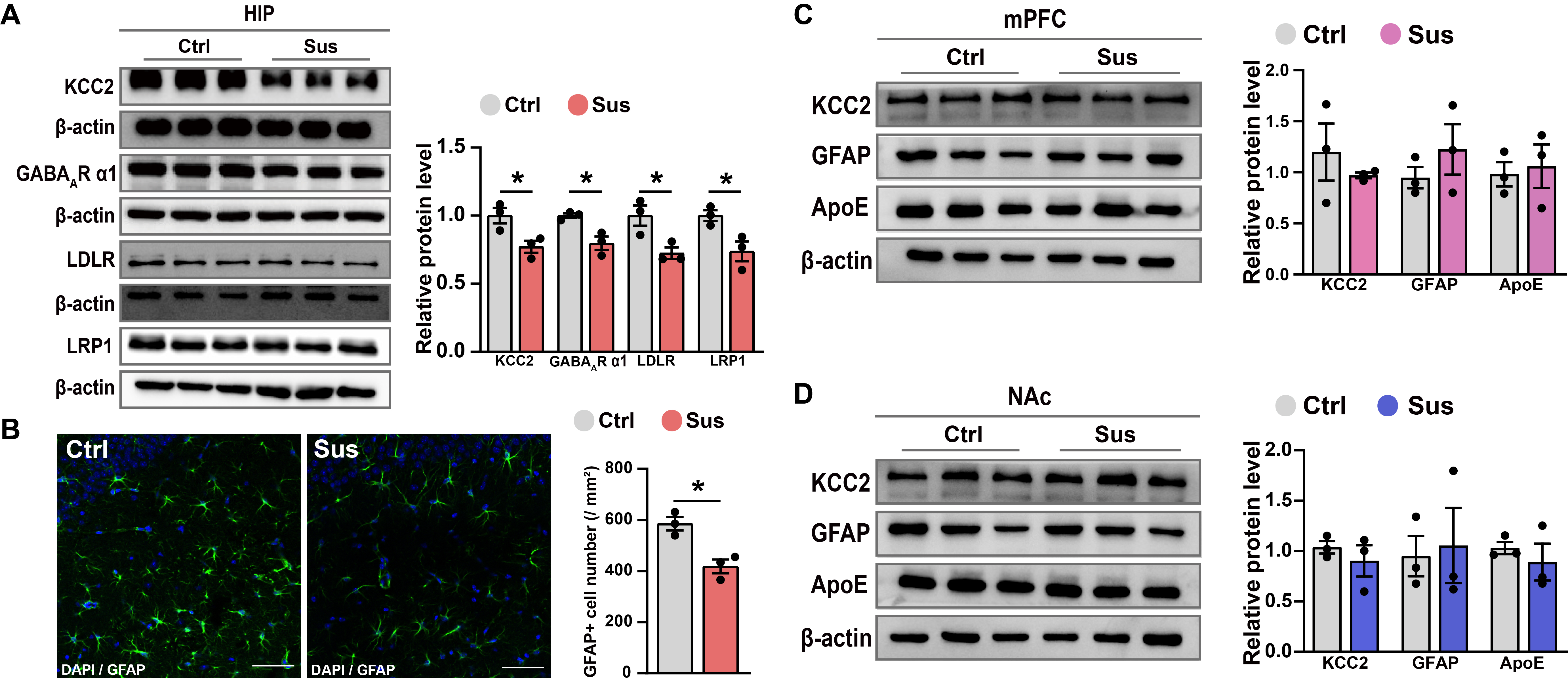


**Supplementary Fig. 1. Decreased protein levels of KCC2, GABA_A_R α1, and ApoE receptors in hippocampus of Sus group mice after CSDS exposure.**

(A) Representative western blot bands for KCC2, GABA_A_R α1, LDLR, and LRP1 in the hippocampus. Statistical results of KCC2, GABA_A_R α1, LDLR, and LRP1 protein levels in the hippocampus (Ctrl, n = 3; Sus, n = 3). (B) Representative images of GFAP immunofluorescence staining in the hippocampus. Statistical results of the number of GFAP+ cells in the hippocampus (Ctrl, n = 3; Sus, n = 3). Scale bar = 20 μm. (C) Representative western blot bands for KCC2, GFAP, ApoE in the mPFC. Statistical results of KCC2, GFAP, ApoE in the mPFC (Ctrl, n = 3; Sus, n = 3). (D) Representative western blot bands for KCC2, GFAP, ApoE in the NAc. Statistical results of KCC2, GFAP, ApoE in the NAc (Ctrl, n = 3; Sus, n = 3). Values are presented as mean ± SEM. Ctrl vs. Sus, **p* < 0.05.


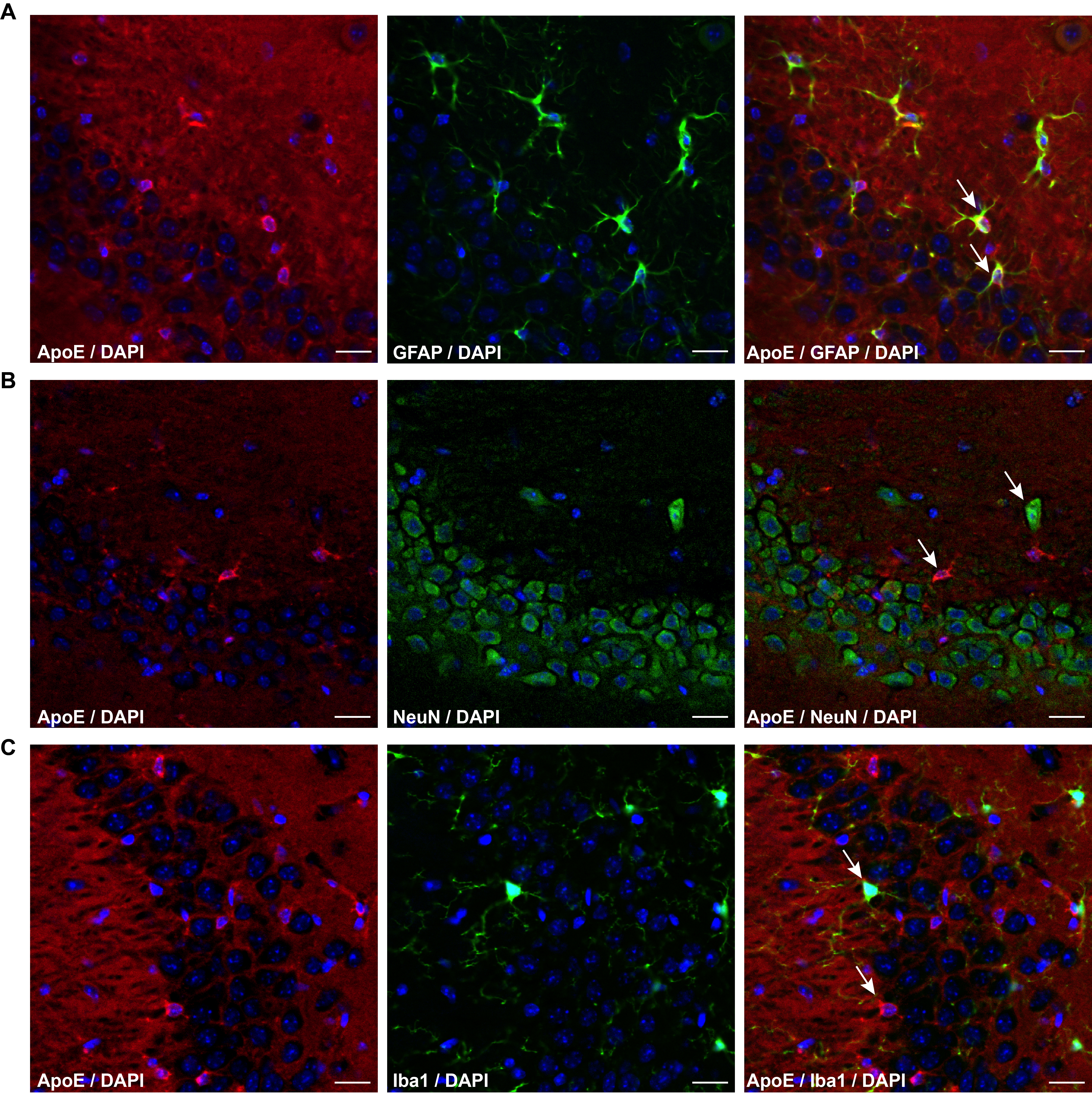


**Supplementary Fig. 2. ApoE and GFAP were co-localized in the hippocampus of C57BL/6J mice.**

(A) The representative fluorescence images of co-localization of GFAP+ and ApoE+ cells in hippocampus of C57BL/6J mice. Scale bar = 20 μm. (B) The representative fluorescence images of NeuN+ and ApoE+ cells in hippocampus of C57BL/6J mice. Scale bar = 20 μm. (C) The representative fluorescence images of Iba1+ and ApoE+ cells in hippocampus of C57BL/6J mice. Scale bar = 20 μm.
